# Intimate partner violence and growth outcomes through infancy: A longitudinal investigation of multiple mediators in a South African birth cohort

**DOI:** 10.1111/mcn.13281

**Published:** 2021-11-03

**Authors:** Whitney Barnett, Raymond Nhapi, Heather J. Zar, Sarah L. Halligan, Jennifer Pellowski, Kirsten A. Donald, Dan J. Stein

**Affiliations:** ^1^ Department of Paediatrics and Child Health, Red Cross War Memorial Children's Hospital, and SA‐Medical Research Council Unit on Child and Adolescent Health University of Cape Town Cape Town South Africa; ^2^ Department of Psychology and Human Development Vanderbilt University Nashville Tennessee USA; ^3^ Department of Psychology University of Bath Bath UK; ^4^ Department of Psychiatry and Mental Health University of Cape Town Cape Town South Africa; ^5^ Department of Behavioral and Social Sciences, School of Public Health Brown University Providence Rhode Island USA; ^6^ Division of Developmental Paediatrics, Department of Paediatrics and Child Health, Red Cross War Memorial Children's Hospital University of Cape Town Cape Town South Africa; ^7^ Neuroscience Institute University of Cape Town Cape Town South Africa; ^8^ South African Medical Research Council Unit on Risk and Resilience in Mental Disorders Cape Town South Africa

**Keywords:** alcohol, infant growth, intimate partner violence, LMIC, mediation, South Africa, tobacco

## Abstract

Intimate partner violence (IPV) has been linked to poor fetal and infant growth. However, factors underlying this relationship are not well understood, particularly in the postnatal time period. In a South African cohort, we investigated (1) associations between IPV in pregnancy and growth at birth as well as postnatal IPV and child growth at 12 months and (2) whether maternal depression, tobacco or alcohol use or infant hospitalizations mediated IPV‐growth relationships. Mothers were enrolled in pregnancy. Maternal IPV was measured during pregnancy and 10 weeks postpartum; depression, alcohol and tobacco use were measured during pregnancy and at 6 months postpartum. Child weight and length were measured at birth and 12 months and converted to z‐scores for analysis. Linear regression and structural equation models investigated interrelationships between IPV and potential mediators of IPV‐growth relationships. At birth, among 1,111 mother–infant pairs, maternal emotional and physical IPV were associated with reduced weight‐for‐age *z*‐scores (WFAZ). Only physical IPV was associated with length‐for‐age *z*‐scores (LFAZ) at birth. Antenatal maternal alcohol and tobacco use mediated IPV‐growth relationships at birth. Postnatally, among 783 mother–infant pairs, emotional and physical IPV were associated with reduced WFAZ at 12 months. Only emotional IPV was associated with LFAZ at 12 months. Maternal tobacco use was a mediator postnatally. Findings highlight the role of physical and emotional IPV as risk factors for compromised fetal and infant growth. Findings underscore the importance of programmes to address interrelated risk factors for compromised infant growth, specifically IPV and substance use, which are prevalent in high‐risk settings.

**Key messages**

- In the current study, both maternal emotional and physical IPV were associated with child growth at birth and 12 months.
- Alcohol and tobacco use emerged as mediators antenatally and tobacco use as a mediator in postnatal IPV‐growth relationships; these may represent an important pathway via which maternal IPV impacts child growth and related interventions should address these.
- Increasing evidence links emotional IPV to adverse child growth; it is important that screening and referral efforts integrate support for emotional IPV alongside physical and sexual IPV.
- IPV remains a highly prevalent public health problem, impacting not only maternal health but child health outcomes, including compromised child growth.

## 1 INTRODUCTION

Growth faltering, a slow rate of gain in a child's weight or height, is an important indicator of poor child health and is prevalent in low‐ and middle‐income country (LMIC) settings. In particular, growth faltering during infancy and early childhood has been linked to adverse short‐ and long‐term health outcomes, including poorer performance in school, delayed child development (Sudfeld et al., 2015), higher frequency and severity of infections (Dewey & Mayers, 2011), increased risk of chronic diseases, including obesity, as well as decreased earnings in adulthood (Adair et al., 2013). Improved understanding of the factors that impact early‐life growth is necessary to develop appropriate interventions that could in turn potentially yield large economic returns and reduce adverse health outcomes through adulthood.

Increasingly, researchers are recognizing the impact of intimate partner violence (IPV) on child growth outcomes. Meta‐analyses have found IPV during pregnancy to increase the risk of low birth weight (Donovan et al., 2016). There is also emerging evidence linking postnatal IPV and growth outcomes in early childhood. Studies have found that IPV is associated with decreased continuous growth outcomes (Sobkoviak et al., 2012) as well as increased risk of stunting, wasting or being underweight for age (Chai et al., 2016; Neamah et al., 2018; Rico et al., 2011; Sobkoviak et al., 2012). Several have been multicountry studies in LMIC settings (Chai et al., 2016; Rico et al., 2011; Smith Fawzi et al., 2019). However, most studies investigated only lifetime IPV or associations using cross‐sectional data. Given that IPV tends to be persistent and is known to affect birthweight, longitudinal analyses of postnatal IPV exposure are essential to further our understanding of postnatal infant growth. Moreover, the majority of studies to date have focused on physical IPV, with very few considering emotional IPV, despite evidence that the latter can also be detrimental to maternal health and well‐being (Chai et al., 2016; Neamah et al., 2018; Ziaei et al., 2014).

Several potential mechanisms through which maternal IPV may impact fetal or infant growth have been proposed. IPV during pregnancy may impact fetal growth directly (e.g., through depression or chronic psychological stress and elevating stress hormones) (Yount et al., 2011) or indirectly, for example, impacting risky behaviour, such as increasing substance use, which in turn may reduce placental blood flow (Shea & Steiner, 2008) or compromise maternal nutrition) (Yount et al., 2011). Postnatally, IPV has been linked to increased maternal depression and substance use (Davis et al., 2017), which may impact a woman's ability to care for her child, for instance, by increasing the risk of child neglect or diverting financial resources from health care costs or nutritionally adequate food (Rahman et al., 2002). Maternal efficacy and financial power within a family are often impacted by abusive relationships, further exacerbating these issues (Shroff et al., 2011). Lastly, children exposed to IPV have been reported to have a higher risk of infection and hospitalizations, both of which may be associated with faltering growth, in particular diarrhoeal diseases (Silverman et al., 2009). Despite this evidence, few studies have formally investigated key mediators in the maternal IPV‐infant growth relationship, especially during the sensitive early postnatal period. This is crucial, as an understanding of the underlying mechanisms, particularly where multiple co‐occurring and prevalent risk factors may be impacting health outcomes, is essential in order to inform interventions that aim to prevent adverse child growth outcomes in association with maternal IPV.

The present study is nested within the Drakenstein Child Health Study (DCHS) a birth cohort study in South Africa, following mother–child dyads from pregnancy through early life with ongoing, detailed follow up including psychosocial risk factor and clinical outcome data. This study therefore provides an opportunity to address key gaps in the literature specifically by (1) investigating IPV and growth using longitudinal data, (2) investigating IPV subtypes in IPV‐growth relationships and (3) exploring the potential role of mediators in these relationships. Previous work in this cohort has shown that physical IPV during pregnancy is associated with low birthweight but did not explore mediators of this relationship (Koen et al., 2014) and that longitudinal postnatal IPV is associated with postnatal depression, which may mediate IPV‐outcome relationships such as child growth (Okafor et al., 2018). We aimed to build upon this work by investigating associations between maternal IPV subtypes (emotional, physical or sexual) and growth outcomes at birth and at 12 months as well as whether maternal depression, alcohol use, tobacco use or infant admission to hospital mediate IPV‐growth relationships.

## 2 METHODS

This study uses data from a birth cohort investigating the early‐life determinants of child health in a peri‐urban area in South Africa (Stein et al., 2015). The parent study collects comprehensive, longitudinal measures of key risk factors across a variety of disciplines (e.g., environmental, infectious, nutritional, genetic, psychosocial, maternal and immunological) that may impact child health.

### 2.1 Setting

The DCHS is located 60 km outside Cape Town, South Africa with a population of approximately 200,000. The area is characterized by low socio‐economic status, low educational attainment and a high proportion of female‐headed households (Stats SA, 2021). Results from the cohort study have also shown high rates of interpersonal violence, substance use and child malnutrition (Barnett et al., 2018; Budree et al., 2017; Myers et al., 2018). This is despite a well‐established, free primary health care system, where more than 90% of the population access antenatal or child health services (Stein et al., 2015; Zar et al., 2015). Support for psychosocial issues and mental health disorders in LMIC settings such as South Africa is limited, with some studies estimating a 90% treatment gap (Demyttenaere et al., 2004). Thus, the psychosocial risk profile of this community may be an important driver of child outcomes such as growth.

### 2.2 Participants

Pregnant women were recruited from two public primary health care clinics, Mbekweni (serving a Black African community) and TC Newman (serving a mixed‐ancestry community). Mothers were enrolled in their second trimester while attending routine antenatal care and have completed assessments antenatally and following birth (ongoing). Women were eligible for the study if they were 18 years or older, between 20 and 28 weeks of gestation, planned attendance at the two recruitment clinics and intended to remain in the area. Data included in the current study were collected antenatally at 28–32 weeks of gestation, at birth, and postnatally at 10 weeks, 6 and 12 months, Figure S1.

### 2.3 Study population

Between March 2012 and March 2015, 1,225 pregnant women were enrolled into the DCHS, as has been described (Zar et al., 2015). Details of study attendance and loss‐to‐follow‐up are provided in Figure S2. A total of 1,111 children had growth outcome data at birth and were included in the antenatal analysis. A total of 783 children who had growth data at 12 months were included in the postnatal analyses, Figure S2.

### 2.4 Growth measures

Infant birth length and weight measurements were conducted by trained labour ward staff, with a subgroup of measurements checked by study staff to confirm reliability. Postnatal anthropometric measurements were done by trained study staff at 12 months. Comprehensive quality control measures were in place including regular training and assessment of staff, routine calibration of equipment and taking multiple measurements. Measurements were performed twice in each child to ensure accuracy. Infant's weight was measured (to the nearest 10 g) in light or no clothing using a Tanita digital platform scale (TAN1584; IL, USA). Recumbent length was measured using a Seca length‐measuring mat (Seca, Hamburg, Germany), performed on a firm surface by two staff members. Equipment was checked and calibrated weekly (Budree et al., 2017). Birth weights and lengths were converted to *z*‐scores based on gender and gestational age using the INTERGROWTH‐21st standards (Villar et al., 2014). Postnatal weight‐for‐age *z*‐scores (WFAZ) and length‐for‐age *z*‐scores (LFAZ) were calculated using weight and length measurements at 12 months, based on age and gender using Anthro software (World Health Organization [WHO], 2006).

### 2.5 Intimate partner violence

The Intimate Partner Violence Questionnaire (IPVQ) is a 12‐item inventory adapted from the WHO multicountry study (Jewkes, 2002) and the Women's Health Study in Zimbabwe (Shamu et al., 2011). The IPVQ has shown high internal consistency and reliability in similar settings and has been widely used in South Africa (Schraiber et al., 2010; Shamu et al., 2016). Further, unpublished data from our cohort have shown a Cronbach's alpha of 0.91, indicating relatively high internal consistency. The IPVQ assessed recent (past‐year) exposure to emotional, physical and sexual abuse. Mothers reported frequency of exposure to partner behaviour (‘never’, ‘once’, ‘a few times’ or ‘many times’). Items were summed to create a total score for each IPV subtype, with higher scores indicating higher frequency and severity of IPV (subtype scores ranged from 3 to 20). Above threshold IPV was defined as more than an isolated event within each subtype, specifically where mothers reported multiple responses of ‘once’ or at least one response of ‘a few times’ or ‘many times’ for each IPV subtype. Mothers completed the IPVQ at the 28‐ to 32‐week antenatal visit and at 10‐week postpartum.

### 2.6 Socio‐demographic and clinical variables

Socio‐demographic variables were collected from a shortened questionnaire used in the South African Stress and Health (SASH) study, a large population‐based study (Zar et al., 2015) and specifically developed for use in a South African setting. Household income and maternal education (any secondary versus completed secondary) were self‐reported antenatally at 28–32 weeks of gestation. Maternal height was measured at enrolment, using a wall‐mounted stadiometer (CE stature meter). Maternal HIV diagnosis was established at enrolment through maternal self‐report and confirmed during routine HIV testing of pregnant women per the Western Cape PMTCT guidelines.

### 2.7 Proposed mediators

Number of hospitalizations included all‐cause child admissions to Paarl Hospital, the only hospital serving the study catchment area. Active surveillance was conducted by study staff at Paarl Hospital. In addition, at routine study visits, mothers were asked whether children had been hospitalized. Where admissions were reported that were missed by surveillance efforts, study staff abstracted relevant details from hospital folders. A score for total number of hospitalizations from 10 weeks through 12 months of child age was calculated for the postnatal analyses.

Validated questionnaires were administered to mothers antenatally and at 6‐month postpartum to assess maternal substance use and depression (Stein et al., 2015). The Alcohol, Smoking and Substance Involvement Screening test (ASSIST), a tool that was developed by the WHO to detect and manage substance use among people attending primary health care services, assessed maternal alcohol and tobacco use risk. It has shown good reliability and validity in international multi‐site studies (Humeniuk et al., 2008) as well as in South Africa (Cronbach's alpha of 0.81 and 0.95 for alcohol and illicit drugs respectively, van der Westhuizen et al., 2016). Previously published results found that self‐reported tobacco use on the ASSIST correlated well with urine cotinine measures, a biomarker of tobacco smoke (Vanker et al., 2017). Individual item responses were summed to generate total scores, with a higher score indicative of greater risk for substance‐related health problems. Scores of 0–10 for alcohol and 0–3 for tobacco have been used to indicate that a participant is at low risk for substance‐related health problems from their current pattern of use; scores of >10 for alcohol and >3 for tobacco indicate moderate or high risk (WHO, 2010). The Edinburgh Postnatal Depression Rating Scale (EPDS) is a 10‐item self‐report measure of recent depressive symptoms (Cox et al., 1987). It has been validated for use in South Africa (de Bruin et al., 2004) and shown high internal consistency (Cronbach's alpha = 0.89). Each item is scored on a frequency scale ranging from 0 to 3, with higher total scores indicative of more severe depressive symptoms. Within a total range possible range of 0 to 30, a cut‐off score of ≥13 was used to indicate probable depression (Cox et al., 1987).

### 2.8 Statistical analysis

Categorical variables were summarized using frequencies and percentages, while continuous variables were summarized using median (interquartile range [IQR]), where not normally distributed. Normality of data was assessed using Shapiro–Wilkes. Mann–Whitney rank sum and Kruskal–Wallis tests were used to test for associations between categorical and continuous variables. Pearson chi‐square test was used to determine if significant associations existed between categorical variables.

To investigate potential mediators of IPV‐growth relationships in this study, we used an iterative approach. Specifically, we used linear regression to identify significant relationships within the proposed models (i.e., Path a, Path b and Path c) prior to formally testing mediation using structural equation models (SEMs). To do this, the following steps were taken. First, we explored bivariate relationships between IPV subtypes and growth outcomes (i.e., total effect; Path c), specifically between antenatal IPV and WFAZ and LFAZ at birth as well as between postnatal IPV and WFAZ and LFAZ at 12 months, and further analyses were only done where these relationships were significant (*p* < 0.05). Second, we investigated bivariate associations between IPV subtypes and each hypothesized mediator (i.e., Path a). Third, we ran adjusted linear regression models, adjusting for covariates and potential mediators to identify mediators that were significantly (*p* < 0.05) associated with growth outcomes considered (i.e., Path b). These relationships were then used to inform criteria, as laid out below, for running formal mediation analyses using SEM. IPV subtypes were included separately in adjusted models due to collinearity. Adjusted linear regression models considered key factors known to affect growth, namely, maternal education, household income, maternal height, maternal HIV status and child sex. Additionally, WFAZ at birth was considered in models investigating growth at 12 months. Covariates were included based on strength of association (*p* value < 0.05) with the growth outcome explored. Final models were estimated using the maximum likelihood method. Variance inflation factor (VIF) was used to check for multicollinearity. These analyses were run using STATA 15.0.

**Figure 1:**
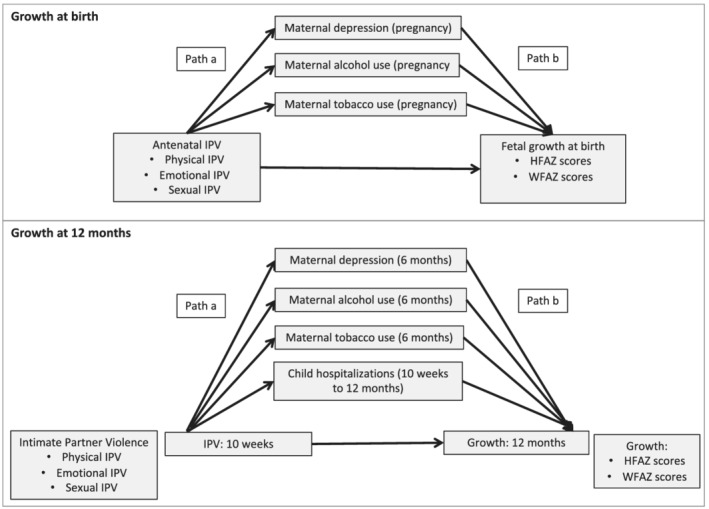
Diagram of hypothesized pathways in the association between intimate partner violence and growth at birth or 12 months

As a final step, multiple mediation analyses were conducted using a SEM approach, which allowed for the estimation of direct and indirect effects, Figure 1. SEMs were used to investigate simultaneously maternal depression, alcohol and/or tobacco use as mediators in the IPV‐growth relationship at birth and at 12 months. Additionally, number of hospitalizations was considered as a potential mediator in IPV‐growth relationships at 12 months. Mediators were tested concurrently (VanderWeele & Vansteelandt, 2014) in final mediation models (1) where IPV sub‐type and the hypothesized mediator were associated (Path a) and (2) where the hypothesized mediator was associated with growth outcomes (*p* < 0.05) in adjusted models (Path b). Hypothesized mediators that did not meet these criteria but were associated with growth outcomes were included as covariates in final models. SEMs were used to estimate the indirect effect of IPV subtypes on growth outcomes via proposed mediators as well as the direct effect of IPV on growth outcomes (Path c′), Figure 1. SEMs were estimated using the maximum likelihood method to impute missing values. All mother–child pairs with anthropometry data at birth or 12 months were included in models of growth at birth and 12 months, respectively. These were conducted using R version 3.6.1 (R Core Team, 2019) and the library ‘lavaan’ version 3.5.3 (Rosseel, 2012). Confidence intervals (95%) as well as direct effects and casual mediation effects were calculated. Model fit was evaluated using root mean square error of approximation (RMSEA; acceptable fit < 0.08) and comparative fit index (CFI > 0.90). Each analysis was based on 5,000 bootstrapped samples (generated from the ‘lavaan’ package version 3.5.3 in R). Proportion mediated was calculated as the indirect effect/total effect and was done using estimations from the SEM models.

### 2.9 Ethical considerations

The DCHS was approved by the Human Research Ethics Committee at the University of Cape Town (401/2009) and by the Western Cape Provincial Health Research committee. Mothers completed informed consent in their preferred language: isiXhosa, Afrikaans or English. Study staff were trained on the ethical conduct of violence research, including confidentiality, mandatory reporting and safety issues. Where substance abuse or mental health issues were identified, staff referred participants to social services or appropriate care in the Paarl area.

## 3 RESULTS

Detailed baseline characteristics are presented in Table 1. The sample was characterized by high levels of above threshold antenatal IPV (34%) and postnatal IPV (27%) as well as a high proportion of stunting at 12 months (15%). Antenatally, 24% of mothers were categorized as depressed, 11% were categorized as moderate or high‐risk for alcohol use, and 28% as moderate or high risk for tobacco use. Similar rates of substance risk were reported postnatally (alcohol, 12%; tobacco, 30%). The majority of mothers did not complete secondary education (61%), and a large portion of households received an income of less than R1,000 per month (USD 60, 38%). There were no differences between the group of children included in the models versus those excluded, Table S1.

**Table 1:** Baseline characteristics of study population

	Antenatal	Postnatal
Sample size^a^	*n* = 1,111	*n* = 783
Socio‐demographic and clinical variables		
Maternal education (did not complete secondary), *n* (%)	676 (61)	486 (62)
Household income, *n* (%)		
<R1,000/month (30 USD)	423 (38)	304 (39)
>R1,000/month	688 (62)	479 (61)
Maternal height (cm), median (IQR)	160 (155, 164)	159 (155, 164)
Male sex, *n* (%)	574 (52)	396 (51)
Mother HIV infected, *n* (%)	241 (22)	173 (22)
Intimate partner violence		
Emotional IPV score, median (IQR)	4 (4, 6)	4 (4, 4)
Emotional IPV threshold, *n* (%)	259 (27)	104 (20)
Physical IPV score, median (IQR)	4 (4, 6)	4 (4, 4)
Physical IPV threshold, *n* (%)	213 (22)	99 (19)
Sexual IPV score, median (IQR)	4 (4, 4)	4 (4, 4)
Sexual IPV threshold, *n* (%)	68 (7)	29 (6)
Any IPV threshold, *n* (%)	328 (34)	141 (27)
Proposed mediators		
Depression score, median (IQR)	9 (6, 12)	8 (5, 10)
Depression threshold, *n* (%)	233 (24)	80 (15)
Alcohol score, median (IQR)	0 (0, 0)	0 (0, 0)
Alcohol: Moderate/high risk	106 (11)	64 (12)
Tobacco score, median (IQR)	0 (0, 13)	0 (0, 12)
Tobacco: Moderate/high risk	274 (28)	158 (30)
Number of hospitalizations, median (IQR)		0 (0, 0)
Any hospitalization, *n* (%)		94 (18)
Growth outcomes		
Weight‐for‐age *z*‐score, median (IQR)	−0.55 (−1.31, 0.07)	−0.04 (−0,89, 0.79)
Length‐for‐age *z*‐score, median (IQR)	0.00 (−0.86, 0.93)	−0.66 (−1.57, 0.19)

*Note*: Above threshold exposure defined as more than an isolated incident within each subtype. Depression threshold defined as EPDS score of ≥13. Moderate/high risk for alcohol was defined as a score of >10; moderate/high risk for tobacco was defined as a score of >3.

Abbreviations: IQR, interquartile range; IPV, intimate partner violence.

^a^ There was missing data for IPV, depression, alcohol and tobacco use (*n* = 134 during pregnancy; *n* = 260 postnatally, where missing these data were imputed in regression and SEM models).

### 3.1 Birth outcomes

In adjusted linear regression analyses, antenatal emotional and physical IPV were inversely associated with WFAZ at birth (emotional IPV *β* = −0.04, 95% CI: −0.07, −0.02 and physical IPV *β* = −0.04, 95% CI: −0.07, −0.02), Table 2. Only physical IPV was associated with LFAZ at birth (*β* = −0.04, 95% CI: 0.08, −0.00), Table 2. Sexual IPV was not associated with either WFAZ or with LFAZ at birth.

**Table 2:** Adjusted associations between intimate partner violence and growth outcomes at birth and through 12 months

	Birth^a^		12 months^b^	
	Weight‐for‐age *z*‐score	Length‐for‐age *z*‐score	Weight‐for‐age *z*‐score	Length‐for‐age *z*‐score
	Coef (95% CI)	Coef (95% CI)	Coef (95% CI)	Coef (95% CI)
Emotional IPV score	−0.04 (−0.07, −0.02)**	−0.03 (−0.07, 0.01)	−0.07 (−0.11, −0.04)**	−0.07 (−0.10, −0.04)**
Physical IPV score	−0.04 (−0.07, −0.02)*	−0.04 (−0.08, −0.00)*	−0.03 (−0.05, −0.00)*	−0.03 (−0.06, 0.01)
Sexual IPV score	−0.05 (−0.13, 0.03)	−0.10 (−0.22, 0.01)	−0.08 (−0.22, 0.07)	−0.10 (−0.31, 0.10)

*Note*: Due to collinearity, each IPV subtype was run in a separate multivariable model. The weight‐for‐age model at birth was adjusted for recruitment site, maternal height and child sex; length‐for‐age model at birth was adjusted for maternal height. Models investigating length‐for‐age and weight‐for‐age at 12 months were adjusted for recruitment site, maternal education, household income, maternal height, child sex and weight‐for‐age *z*‐scores at birth. IPV subtypes were included as continuous variables in all models.

^a^ Sample size at birth, *n* = 972.

^b^ Sample size at 12 months, *n* = 783.

* *p* < 0.05.

** *p* < 0.001.

### 3.2 Mediation models: Birth size

Prior to running formal mediation models, linear regression analyses were run to test for associations between IPV subtypes and hypothesized meditators (Path a, Table S3) and between hypothesized mediators and growth outcomes (Path b, Table S2). Both emotional and physical IPV were associated with maternal alcohol and tobacco use and depression during pregnancy (Path a). However, only alcohol and tobacco use during pregnancy were associated with WFAZ and LFAZ at birth (in both emotional and physical IPV analyses, Path b), Table S2. These were therefore formally investigated as mediators. Maternal antenatal depression was not associated with birth size and therefore was not included as a covariate in final models. Details about variable selection for model building appear in Table S4; variables were controlled for in final models based on significant (*p* < 0.05) bivariate relationships with the outcome explored.

**Figure 2:**
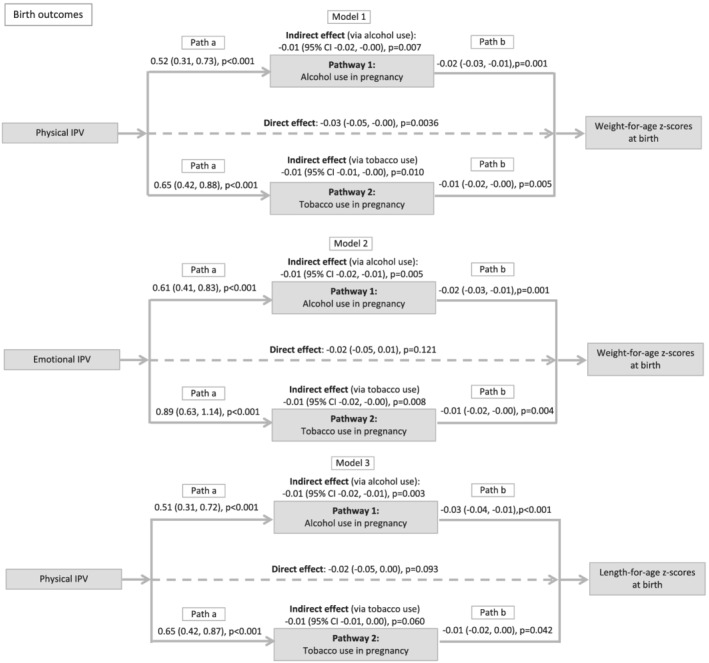
Results of structural equation models investigating mediators of intimate partner violence and growth at birth. *Note*: The figure shows results (regression path coefficients, 95% confidence intervals and *p* values) for each direct and indirect association. Structural equation models were used to investigate potential mediators in the relationship between antenatal intimate partner violence (IPV) and growth at birth. Models 1 and 2: Adjusted for maternal height and child sex. Model 3: Adjusted for maternal height

We examined alcohol and tobacco use as mediators of the physical IPV‐WFAZ association at birth (Model 1), the emotional IPV‐WFAZ association at birth (Model 2), and the physical IPV‐LFAZ association at birth (Model 3). In Model 1, the relationship between antenatal physical IPV and WFAZ was partially mediated by tobacco and alcohol use (total proportion mediated = 39%). The direct effect of physical IPV on WFAZ remained significant after including potential mediators and confounders. In Model 2, the relationship between antenatal emotional IPV and WFAZ at birth was fully mediated by tobacco and alcohol use (total proportion mediated = 51%). In Model 3, the relationship between antenatal physical IPV and LFAZ at birth was fully mediated by alcohol use in pregnancy (proportion mediated = 36%). Tobacco use did not mediate this relationship. Results are summarized in Figure 2.

### 3.3 Growth at 12 months

In adjusted analyses, emotional and physical IPV were inversely associated with WFAZ at 12 months (emotional IPV *β* = −0.07, 95% CI: −0.11, −0.04; physical IPV *β* = −0.03, 95% CI: −0.05, −0.00), Table 2. Only emotional IPV was associated with reduced LFAZ at 12 months (*β* = −0.07, 95% CI: −0.10, −0.04).

### 3.4 Mediation models: Growth at 12 months

Initial steps of the analytic approach tested associations between IPV subtypes and hypothesized mediators (Path a, Table S3) as well as between hypothesized mediators and growth outcomes (Path b, Table S2) using linear regression models. Of proposed mediators for postnatal IPV‐growth relationships, only postnatal tobacco use was associated with growth outcomes at 12 months (WFAZ and LFAZ, Path b) and with IPV (Path a), Table S2. Therefore, postnatal tobacco use was formally investigated as a mediator. Details about modelling building and variable selection appear in Table S4. Variables were controlled for in final models based on significant (*p* < 0.05) bivariate relationships with the outcome explored. Postnatal alcohol use was associated with WFAZ and LFAZ in bivariate analyses (Path b), but maternal depression and child hospitalizations were not. Therefore, alcohol use was included as a covariate but not investigated as a mediator. Both postnatal emotional and physical IPV were associated with maternal depression, alcohol use and tobacco use but not child hospitalizations (Path a).

**Figure 3:**
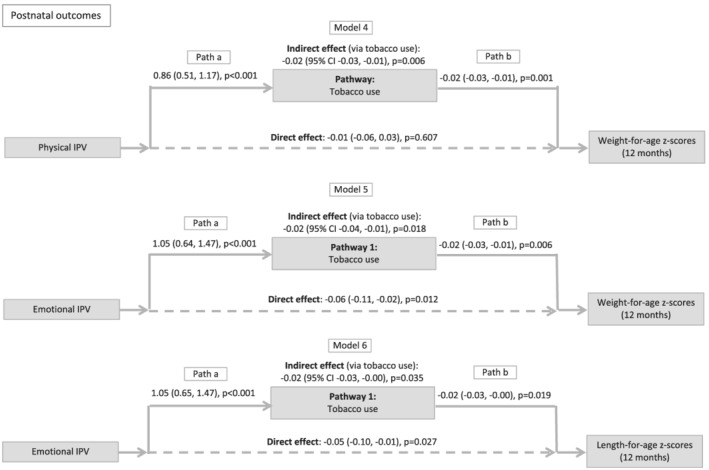
Results of structural equation models investigating mediators of intimate partner violence and growth at 12 months. *Note*: The figure shows results (regression path coefficients, 95% confidence intervals and *p* values) for each direct and indirect association. Structural equation models were used to investigate potential mediators in the relationship between postnatal intimate partner violence (IPV) and growth at 12 months. All models adjusted for maternal height, child sex, household income, weight for age *z*‐score at birth, maternal education and alcohol use

We examined postnatal tobacco use as a mediator of the physical IPV‐WFAZ association at 12 months (Model 4), the emotional IPV‐WFAZ association (Model 5), and the emotional IPV‐LFAZ association at 12 months (Model 6). In Model 4, the relationship between postnatal physical IPV and WFAZ was fully mediated by tobacco use (proportion mediated = 62%). In Model 5, the relationship between postnatal emotional IPV and WFAZ at 12 months was partially mediated by tobacco use (proportion mediated = 23%). In Model 6, the relationship between postnatal emotional IPV and LFAZ at 12 months was partially mediated by tobacco use (proportion mediated = 24%). Emotional IPV maintained a direct effect on WFAZ and LFAZ at 12 months. Results are summarized in Figure 3.

## 4 DISCUSSION

In this study, investigating maternal IPV and infant growth outcomes in a South African birth cohort, we describe high prevalence of both maternal antenatal and postnatal IPV. At birth, both maternal emotional and physical IPV were associated with lower WFAZ, with physical IPV associated with lower LFAZ at birth. Maternal alcohol use and tobacco use during pregnancy mediated the relationship between maternal IPV and WFAZ, whereas alcohol use mediated the relationship between physical IPV and LFAZ at birth. Postnatally, both maternal emotional and physical IPV were associated with lower infant WFAZ at 12 months of age, whereas emotional IPV was associated with lower child LFAZ at 12 months of age. Postnatal maternal tobacco use mediated these relationships. Findings build on previous research by investigating IPV's impact on child growth through infancy and by formally testing multiple mediators collected in a birth cohort study.

Our results linking IPV during pregnancy with growth at birth is consistent with previous literature; specifically associations with low birth weight are well‐established (Donovan et al., 2016). Both smoking and alcohol use during pregnancy have also been well documented as factors associated with fetal growth (Patra et al., 2011; Pereira et al., 2017). Though many studies have investigated IPV, substance use and intrauterine growth, few have formally tested mediation. A US study investigating mediators of emotional or physical IPV and birthweight found that both forms of IPV were associated with lower birthweight and that tobacco use mediated this relationship (Kearney et al., 2004). Tobacco and alcohol use often co‐occur, increasing the potential impact on pregnancy outcomes. Importantly, pregnant women who smoke often sustain similar rates of tobacco use postnatally (Fitzpatrick et al., 2016), which may continue to impact child health and growth outcomes. Our findings support research linking IPV to substance use during pregnancy as well as growth restriction in utero; they also extend this research to an LMIC context and formally investigate substance use as a mediator in this relationship,

In the postnatal period, the association between emotional IPV and compromised infant growth is a novel finding. We found that both maternal physical and emotional IPV are associated with reduced child WFAZ at 12 months and that there is an association between recent emotional IPV and child LFAZ at 12 months. In contrast, a study, in Brazil, investigated past‐year maternal emotional or physical abuse, found severe physical and not verbal or less severe physical abuse, to be associated with child WFAZ in the first 2 years of life (Hasselmann & Reichenheim, 2006). Another study that investigated emotional IPV found no association with linear growth (Sobkoviak et al., 2012). The existing literature investigating postnatal associations between IPV and growth are relatively few and use a wide range of definitions for IPV exposure with the majority investigating lifetime IPV rather than recent exposure. These novel findings, related to postnatal maternal emotional IPV and early child growth outcomes, require replication in additional cohorts.

Maternal smoking mediated the IPV‐growth relationship at 12 months of age, after adjusting for clinical and socio‐demographic variables. Exposure to tobacco smoke has been linked to poor child growth, in particular, reduced linear height as well as stunting (Nadhiroh et al., 2020). It can be difficult to distinguish causal effects in the presence of potential unmeasured environmental and socio‐economic factors. For instance, women who smoke postnatally are more likely to have smoked during pregnancy, a known risk factor for fetal growth restriction, which is known to impact subsequent growth; we were therefore careful to adjust for birthweight in final models. Potential reasons for the association between smoke exposure and poor infant growth include family income diverted from the purchase of nutritious food to cigarettes (Best et al., 2007) and that smoke exposure has been shown to cause frequent health problems in children, which may additionally impact growth (Control, 2019).

To date, only one study investigating IPV and postnatal growth has formally investigated potential mediators (Neamah et al., 2018). This study examined multiple potential mediators including alcohol use, positive–negative cognitive stimulation, depression and number of adults and/or children in the IPV‐stunting relationship did not find evidence of mediation for any factors investigated. Other studies have adjusted for potential mediators but did not formally investigate mediation. For example, one study included recent child illness as a potential confounder, finding that physical and sexual IPV were significantly associated with child stunting and underweight, after adjusting for childhood illness (Rahman et al., 2012). Another study proposed maternal care seeking behaviour but found that this did not substantially change the association between maternal IPV and infant growth outcomes in the adjusted model (Rico et al., 2011). Further work is needed to replicate findings in the current study showing that tobacco use mediated IPV‐growth relationships postnatally. It is important that future research aims to understand the underlying reasons for this (e.g., diverted income or health problems related to smoke exposure) to inform interventions.

### 4.1 Policy implications

The majority of women in South Africa as well as worldwide attend antenatal care, offering an opportunity to screen for maternal IPV as well as substance use and refer for help. Public health efforts have made huge strides in informing mothers of the potential negative child health sequelae of alcohol and tobacco use, specifically during pregnancy. However, this has also created stigma, establishing a barrier to disclosure or treatment (Stone, 2015). Existing programmes have also tended to focus on educating mothers about the dangers of alcohol or tobacco smoke exposure antenatally without including a focus on the postnatal period. Further, it is important that screening and referral efforts integrate support for emotional IPV alongside physical and sexual IPV, given its potential impact on both substance use as well as child growth.

### 4.2 Strengths and limitations

Inclusion criteria for the parent study were broad; however, study recruitment was done during routine antenatal care and mothers who did not present or presented very late for antenatal care were not included. This may have resulted in the highest risk mothers being underrepresented; however, this would likely have strengthened findings. Key mediators were also self‐reported, specifically maternal depression, alcohol and tobacco use, which may have been underreported, particularly in pregnancy given the stigma, in particular relating to alcohol use perinatally. Some instances of child hospitalizations may have been missed where not reported by mothers or if they occurred outside the study area; however, active surveillance systems were strong to capture these. Postnatal IPV was measured at 10 weeks and may therefore overlap antenatal IPV exposure; however, postnatal IPV was measured at multiple points postnatally and found to be correlated with stable intensity profiles through 2‐year postpartum (Barnett et al., 2018). In addition, there were children who were not included in analyses due to incomplete data, although there was no evidence that these excluded children differed from the wider sample on key risk factors. Lastly, IPV subtypes were analysed separately due to collinearity; however, these co‐occur to a large degree and disentangling subtype‐specific effects is difficult and should be interpreted with caution. Despite these limitations, the current study adds to a small growing literature investigating IPV and growth outcomes postnatally. Further, we address several methodological gaps in the existing literature, specifically by using longitudinal data, considering recent rather than lifetime IPV and including emotional IPV as a key exposure, rather than defining IPV as only physical and/or sexual.

### 4.3 Data sharing

Collaborations from external researchers are welcome. The study has a large and active group of investigators and postgraduate students with a track record of successful partnership with researchers or students from other institutions. Researchers who are interested in collaborations or access to study datasets can find additional information on our website http://www.paediatrics.uct.ac.za/scah/dclhs.

## CONFLICTS OF INTEREST

The authors declare that they have no conflicts of interest.

## CONTRIBUTIONS

WB and SLH conceived the study and WB conducted the data analysis with support from RN and JP. HJZ is the principal investigator of the DCHS and DJS leads the psychosocial aspects. WB drafted the manuscript; all authors reviewed, contributed to and approved the final manuscript.

## Supporting information


**Figure S1.** Timing of measures

**Figure S2.** Flow diagram of attendance and loss to follow up from enrolment through the first year of life.
**Table S1.** Comparison of psychosocial, demographic and clinical data between child observations included in final models and those not included.
**Table S2.** Mediator adjusted associations between intimate partner violence and growth outcomes at birth and through 12 months.
**Table S3.** Unadjusted associations between intimate partner violence and proposed mediators.
**Table S4.** Unadjusted associations between covariates, intimate partner violence and mediators and infant weight‐for‐age z‐scores and length‐for‐age z‐scores at birth

## Data Availability

Collaborations from external researchers are welcome. The study has a large and active group of investigators and postgraduate students with a track record of successful partnership with researchers or students from other institutions. Researchers who are interested in collaborations or access to study datasets can find additional information on our website [blinded].
