# Supplementary material for: Intimate partner violence and growth outcomes through infancy: A longitudinal investigation of multiple mediators in a South African birth cohort
Source: Matern Child Nutr. 2021 Nov 3;18(1):e13281. doi: 10.1111/mcn.13281 (PMC8710113; doi:10.1111/mcn.13281)
Supplement: Supplementary file 1 — Figure S1. Timing of measures [file MCN-18-e13281-s002.pdf]

Supplemental Figure 1. Timing of measures

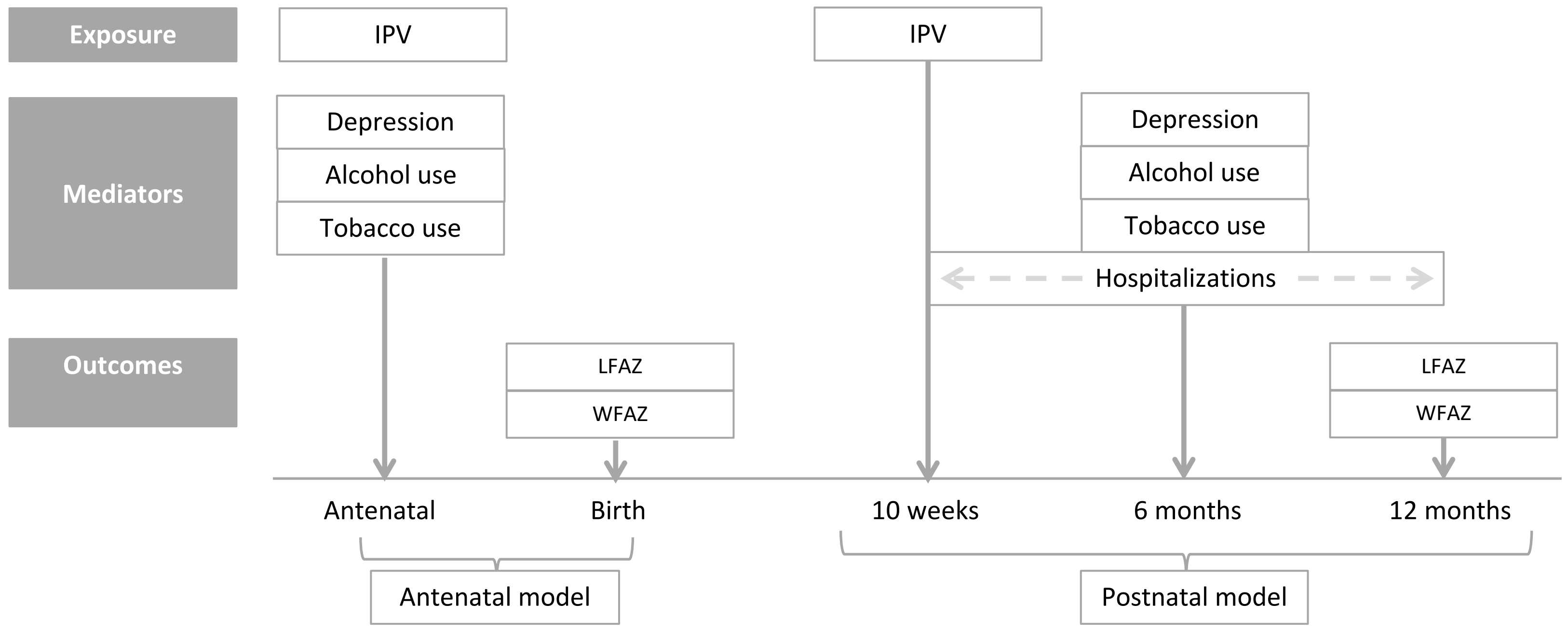

Time points of measures included in the current study. IPV= Intimate partner violence (emotional, physical and sexual); LFAZ = length-for-age z-scores; WFAZ = weight-for-age z-scores; Child hospitalizations were measured by active surveillance and included as total number of hospitalizations per child from age 10 weeks to 12 months.
