# Supplementary material for: Intimate partner violence and growth outcomes through infancy: A longitudinal investigation of multiple mediators in a South African birth cohort
Source: Matern Child Nutr. 2021 Nov 3;18(1):e13281. doi: 10.1111/mcn.13281 (PMC8710113; doi:10.1111/mcn.13281)
Supplement: Supplementary file 2 — Figure S2. Flow diagram of attendance and loss to follow up from enrolment through the first year of life. Table S1. Comparison of psychosocial, demographic and clinical data between child observations included in final models and those not included. Table S2. Mediator adjusted associations between intimate partner violence and growth outcomes at birth and through 12 months. Table S3. Unadjusted associations between intimate partner violence and proposed mediators. Table S4. Unadjusted associations between covariates, intimate partner violence and mediators and infant weight‐for‐age z‐scores and length‐for‐age z‐scores at birth [file MCN-18-e13281-s001.pdf]

Supplemental Figure 2. Flow diagram of attendance and loss to follow up from enrolment through the first year of life.

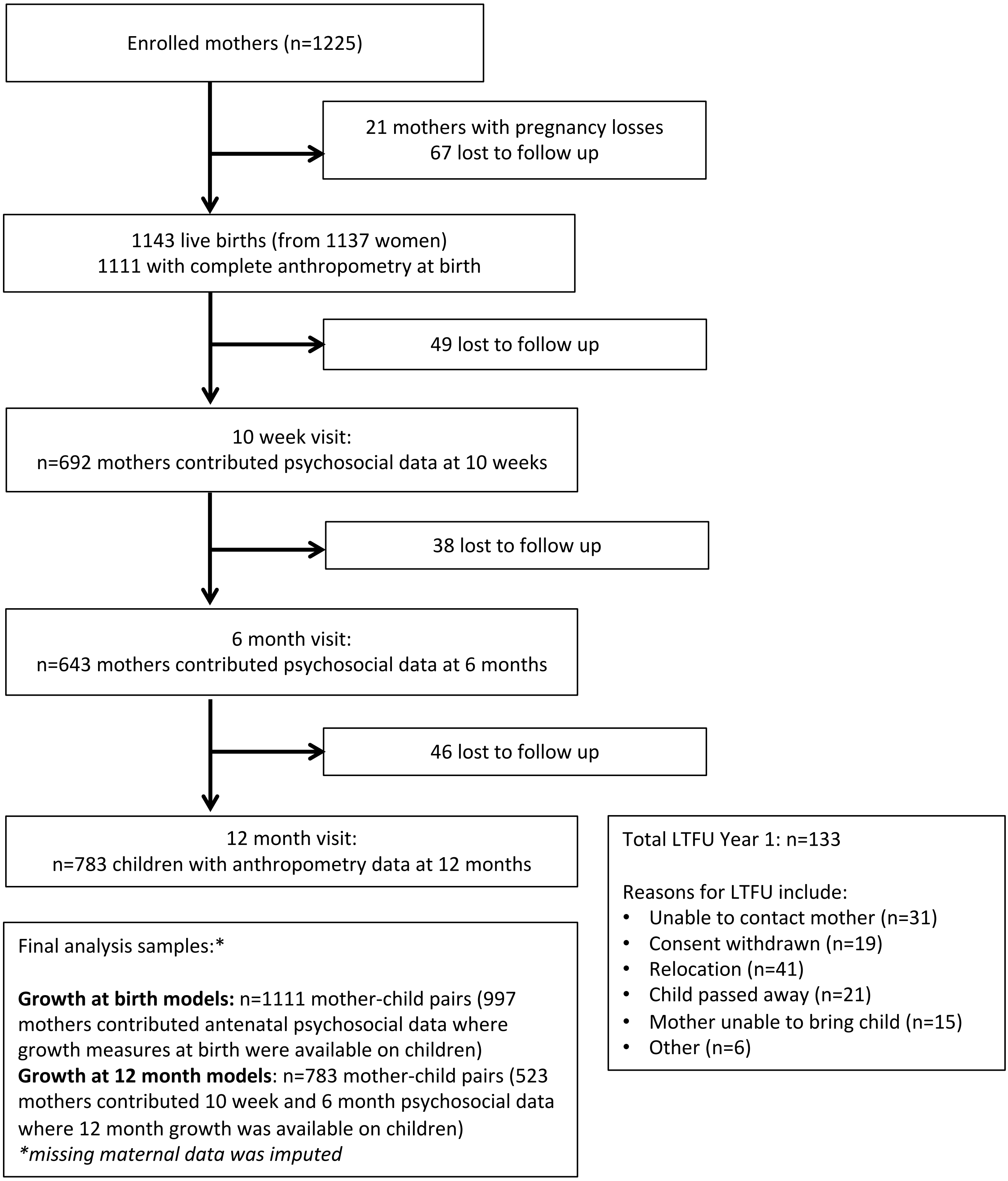

Supplemental Information

Supplemental Table 1. Comparison of psychosocial, demographic and clinical data between child observations included in final models and those not included.

	In model	Not in model	p-value
Sociodemographic & clinical variables			
Sample	*n=783*	*n=352*	
Education: did not complete secondary education, n (%)	486 (62)	205 (58)	0·204
Household income:			
<R1,000, n (%)	479 (61)	226 (64)	0·330
Maternal Height (cm), median (IQR)	159·1 (155, 164)	159 (155, 164)	0·616
Gender: Male, n (%)	396 (51)	186 (53)	0·502
WAZ at birth, median (IQR)	-0·55 (-1·32, 0·11)	-0·53 (-1·19, -0·01)	0·711
Intimate Partner Violence (antenatal)			
Sample	*n=977*	*n=15*	
Emotional IPV score, median (IQR)	4 (4, 6)	4 (4, 7)	0·472
Physical IPV score, median (IQR)	4 (4, 6)	4 (4, 6)	0·947
Sexual IPV score, median (IQR)	4 (4, 4)	4 (4, 4)	0·548
Intimate Partner Violence (10 weeks)			
Sample	*n=523*	*n=169*	
Emotional IPV score , median (IQR)	4 (4, 4)	4 (4, 4)	0·921
Physical IPV score, median (IQR)	4 (4, 4)	4 (4, 4)	0·544
Sexual IPV score, median (IQR)	4 (4, 4)	4 (4, 4)	0·694
Mediator variables (Antenatal)			
Sample	*n=977*	*n=15*	
Depression score, median (IQR)	9 (6, 12)	8 (7, 10)	0·761
Alcohol score, median (IQR)	0 (0, 0)	0 (0, 6)	0·507
Tobacco score, median (IQR)	0 (0, 13)	0 (0, 9)	0·817
Mediator variables (6 months)			
Sample	*n=523*	*n=118*	
Depression score, median (IQR)	8 (5, 10)	7 (5, 10)	0·976
Alcohol score, median (IQR)	0 (0, 0)	0 (0, 2)	0·580
Tobacco score, median (IQR)	0 (0, 12)	0 (0, 0)	0·059
Sample	*n=783*	*n=352*	
Child hospitalizations (10 weeks to 12 months)	0 (0, 0)	0 (0, 0)	0·175

Supplemental Table 2. Mediator adjusted associations between intimate partner violence and growth outcomes at birth and through 12 months.

**Emotional IPV Models**				
	**Birth^**		**Through 12 months#**	
	Weight-for-age z-scores Coef (95% CI)	Length-for-age z-scores Coef (95% CI)	Weight-for-age z-scores Coef (95% CI)	Length-for-age z-scores Coef (95% CI)
Intimate Partner Violence				
Emotional IPV score	-0·02 (-0·05, 0·00)	-0·01 (-0·04, 0·03)	-0·06 (-0·09, -0·03)**	-0·05 (-0·10, -0·00)*
Proposed Mediators				
Alcohol risk score	-0·02 (-0·03, -0·01)**	-0·03 (-0·04, -0·01)*	0·00 (-0·01, 0·01)	-0·01 (-0·03, 0·01)
Tobacco risk score	-0·01 (-0·02, -0·00)*	-0·01 (-0·02, -0·00)*	-0·02 (-0·03, -0·01)*	-0·02 (-0·03, -0·00)*
**Physical IPV Models**				
	**Birth^**		**Through 12 months#**	
	Weight-for-age z-scores Coef (95% CI)	Length-for-age z-scores Coef (95% CI)	Weight-for-age z-scores Coef (95% CI)	Length-for-age z-scores Coef (95% CI)
Intimate Partner Violence				
Physical IPV score	-0·03 (-0·05, -0·00)*	-0·02 (-0·04, -0·01)*	-0·01 (-0·05, 0·03)	-0·01 (-0·04, 0·03)
Proposed Mediators				
Alcohol risk score	-0·02 (-0·03, -0·01)**	-0·02 (-0·04, -0·01)*	-0·00 (-0·01, 0·02)	-0·01 (-0·02, 0·00)
Tobacco risk score	-0·01 (-0·02, -0·00)*	-0·01 (-0·02, -0·00)*	-0·02 (-0·04, -0·01)*	-0·02 (-0·03, -0·01)*

Notes: Mediator adjusted models were run to confirm association (p<0·05) between proposed mediator and growth outcome prior to running formal mediation models. Due to collinearity, emotional and physical IPV were run in separate multivariable models. Weight-for-age model at birth was adjusted for recruitment site, maternal height and child sex; length-for-age model at birth was adjusted for maternal height. Models investigating length-for-age and weight-for-age at 12 months were adjusted for recruitment site, maternal education, household income, maternal height, child sex and weight-for-age z-scores at birth). IPV sub-types, alcohol and tobacco use are included as continuous variables.

*p<0·05; **p<0·001

^Sample size at birth, n=972; #Sample size at 12 months, n=783

Supplemental Table 3. Unadjusted associations between intimate partner violence and proposed mediators.

Antenatal			
	Depression score<br>Coefficient (95% CI)	Alcohol score<br>Coefficient (95% CI)	Tobacco score<br>Coefficient (95% CI)
Emotional IPV	0·12 (0·06, 0·18)**	0·09 (0·06, 0·13)**	0·07 (0·05, 0·09)**
Physical IPV	0·14 (0·10, 0·19)**	0·10 (0·05, 0·15)**	0·06 (0·04, 0·08)**

Postnatal				
	Depression score<br>Coefficient (95% CI)	Alcohol score<br>Coefficient (95% CI)	Tobacco score<br>Coefficient (95% CI)	Child hospitalisations<br>Coefficient (95% CI)
Emotional IPV	0·26 (0·15, 0·56)*	0·73 (0·42, 1·04)**	0·98 (0·61, 1·35)**	-0·00 (-0·01, 0·01)
Physical IPV	0·24 (0·06, 0·41)*	0·77 (0·52, 1·03)**	0·78 (0·47, 1·09)**	0·00 (-0·01, 0·01)

Associations explored by linear regression. IPV sub-types, depression, alcohol and tobacco use are included as continuous variables.
*p<0·05; **p<0·001

Supplemental Table 4. Unadjusted associations between covariates, intimate partner violence and mediators and infant weight-for-age z-scores and length-for-age z-scores at birth

	Weight-for-age z-scores Unadjusted Coef (95% CI)	Length-for-age z-scores Unadjusted Coef (95% CI)
Intimate Partner Violence (antenatal)		
Emotional IPV score	-0·04 (-0·06, -0·02)*	-0·03 (-0·07, -0·00)*
Physical IPV score	-0·04 (-0·06, -0·02)**	-0·04 (-0·07, -0·01)*
Sexual IPV score	-0·06 (-0·14, 0·01)	-0·12 (-0·22, 0·02)
Sociodemographic & clinical variables		
Education: did not complete secondary education	-0·10 (-0·24, 0·03)	-0·17 (-0·34, 0·01)
Household income:		
<R1,000	0·03 (-0·11, 0·17)	0·07 (-0·11, 0·25)
Maternal Height (cm)	0·02 (0·01, 0·03)**	0·02 (0·01, 0·03)*
Gender: Male	-0·17 (-0·31, -0·04)*	-0·17 (-0·34, 0·01)
HIV exposed	0·09 (-0·07, 0·26)	-0·01 (-0·22, 0·20)
Mediator variables (antenatal)		
Depression score	-0·01 (-0·02, 0·00)	-0·00 (-0·02, 0·01)
Alcohol score	-0·02 (-0·03, -0·02)**	-0·03 (-0·04, -0·02)**
Tobacco score	-0·02 (-0·02, -0·01)**	-0·02 (-0·02, -0·01)**

Associations explored by linear regression. IPV sub-types, depression, alcohol and tobacco use are included as continuous variables.
*p<0·05; **p<0·001

Supplemental Table 5. Unadjusted associations between covariates, intimate partner violence, proposed mediators and infant growth at 12 months.

	Weight-for-age z-scores (12 months) Unadjusted Coef (95% CI)	Length-for-age z-scores (12 months) Unadjusted Coef (95% CI)
Intimate Partner Violence (10 weeks)		
Emotional IPV Score	-0·10 (-0·14, -0·06)**	-0·10 (-0·14, -0·05)**
Physical IPV Score	-0·05 (-0·09, -0·01)*	-0·05 (-0·09, -0·01)*
Sexual IPV Score	-0·14 (-0·31, 0·04)	-0·15 (-0·33, 0·03)
Sociodemographic & clinical variables		
Education: did not complete secondary education	-0·48 (-0·66, -0·29)**	-0·50 (-0·69, -0·31)**
Household income:		
<R1,000	-0·19 (-0·37, 0·00)	-0·26 (-0·45, -0·07)*
Maternal Height (cm)	0·04 (0·03, 0·05)**	0·04 (-0·02, 0·05)**
Gender: Male	-0·32 (-0·50, -0·14)*	-0·45 (-0·64, -0·27)**
WAZ at birth	0·33 (0·26, 0·41)**	0·27 (0·19, 0·35)**
HIV exposed	0·16 (-0·06, 0·38)	0·01 (-0·22, 0·23)
Mediator variables (6 months)		
Hospitalizations	-0·09 (-0·40, 0·23)	-0·07 (-0·39, 0·25)
Depression score	-0·00 (-0·02, 0·02)	0·00 (-0·02, 0·02)
Alcohol score	-0·02 (-0·03, -0·00)*	-0·02 (-0·04, -0·01)*
Tobacco score	-0·03 (-0·04, -0·02)**	-0·03 (-0·04, -0·01)**

Associations explored by linear regression. IPV sub-types, depression, alcohol and tobacco use are included as continuous variables.
*p<0·05; **p<0·001
